# Supplementary material for: A CPC-shelterin-BTR axis regulates mitotic telomere deprotection
Source: Nat Commun. 2025 Mar 17;16:2277. doi: 10.1038/s41467-025-57456-8 (PMC11914695; doi:10.1038/s41467-025-57456-8)

SUPPLEMENTARY INFORMATION

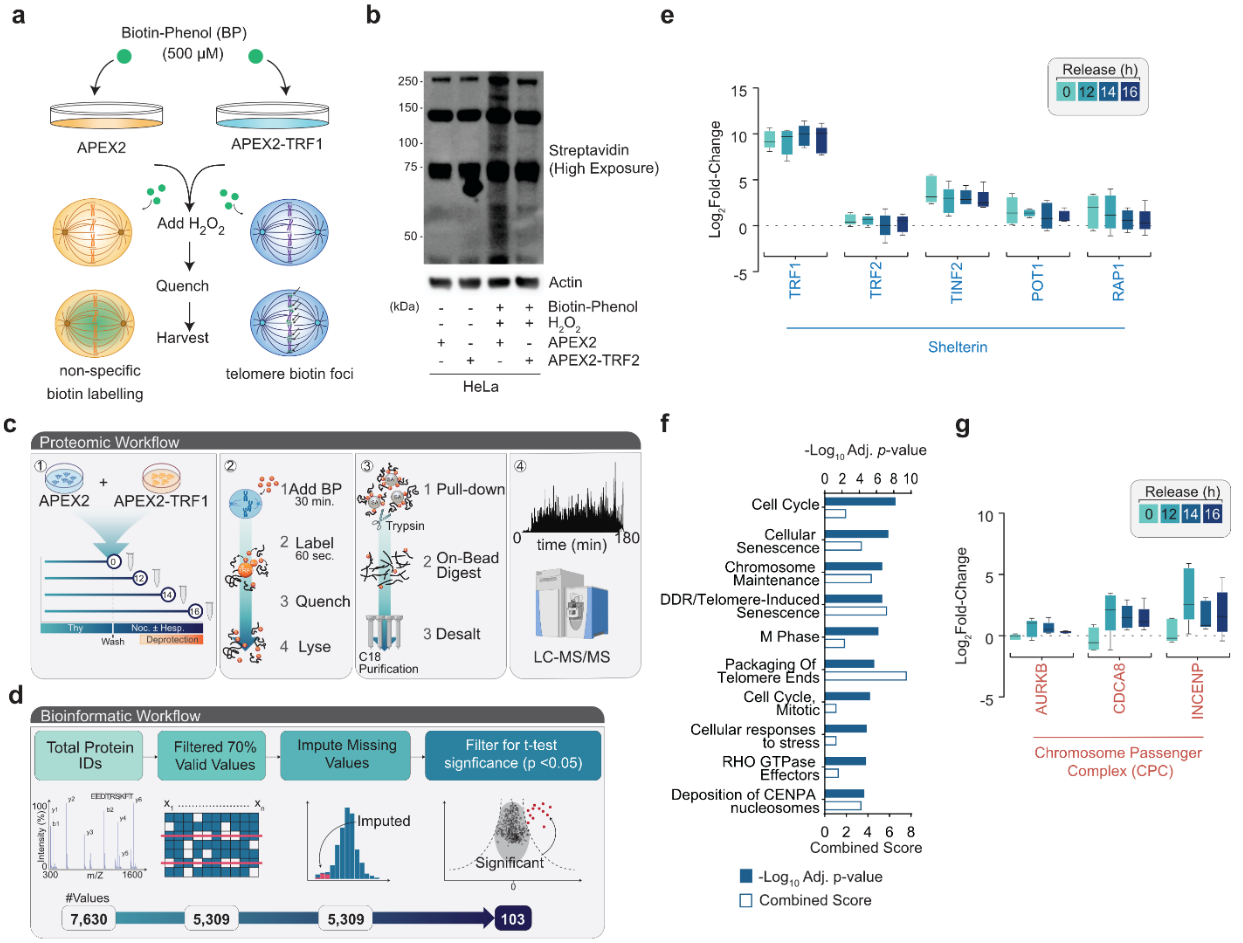

Supplementary Figure 1. Proximity dependent TRF1 interactomics reveals components of the MAD telomere deprotection pathway. a. Schematic of APEX2-TRF1 proximal biotin-labelling for interactomics. **b.** Immunoblot of streptavidin bound to whole cell extracts derived from APEX2 or APEX2-TRF1 HeLa cells with or without APEX2 activation with Biotin-phenol and hydrogen peroxide (H_2O_2) (representative example of n = 3 biological replicates). **c, d.** Schematic of the proteomics (c) and bioinformatics (d) workflow for APEX2-TRF1 interactomics. Drawing of the mass spectrometer was created in BioRender (Cesare, T. (2025) https://BioRender.com/r86h834). **e.** Log_2-fold change in streptavidin-precipitated shelterin proteins between APEX2-TRF1 and APEX2 samples (Tukey's boxplot, median ± IQR, n = 5 biological replicates). **f.** Enrichr gene ontology analysis of proteins significantly enriched within mitotic Flag-APEX2-TRF1 datasets. Ontology terms are ranked by $-Log_{10}$ p-value (filled bars), reporting Enrichr combined score (empty bars). **g.** Log_2-fold change in streptavidin-precipitated Chromosome Passenger Complex proteins between APEX2-TRF1 and APEX2 samples (Tukey's boxplot, median ± IQR, n = 5 biological replicates). Quantitative source data are provided as a Source Data file and source blots at the end of this document.

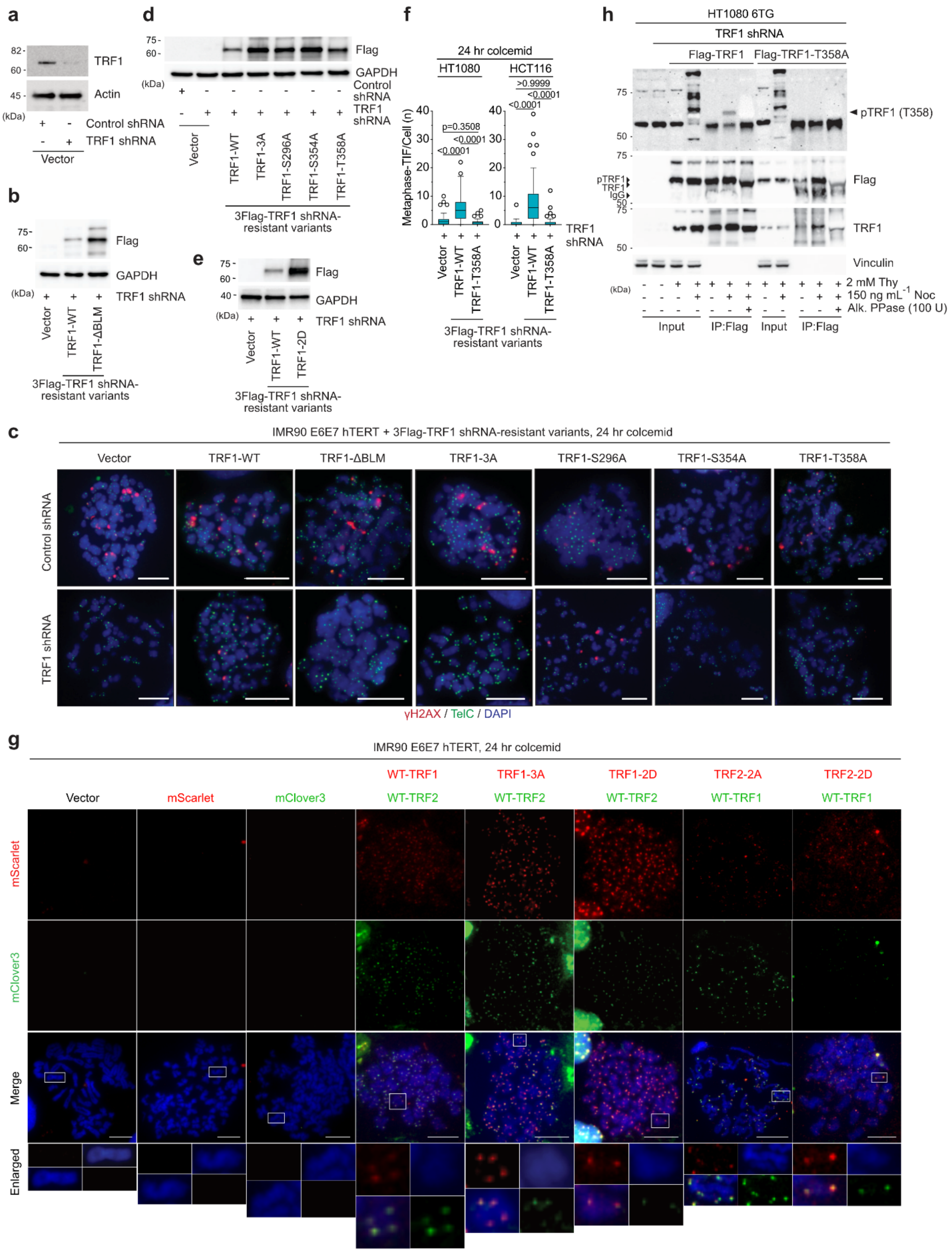

a
TRF1
Actin
Control shRNA
TRF1 shRNA
Vector
(kDa)
b
Flag
GAPDH
TRF1 shRNA
Vector
TRF1-WT
TRF1-ΔBLM
3Flag-TRF1 shRNA-resistant variants
c
IMR90 E6E7 hTERT + 3Flag-TRF1 shRNA-resistant variants, 24 hr colcemid
Vector
TRF1-WT
TRF1-ΔBLM
TRF1-3A
TRF1-S296A
TRF1-S354A
TRF1-T358A
Control shRNA
TRF1 shRNA
γH2AX / TelC / DAPI
d
Flag
GAPDH
Control shRNA
TRF1 shRNA
Vector
TRF1-WT
TRF1-3A
TRF1-S296A
TRF1-S354A
TRF1-T358A
3Flag-TRF1 shRNA-resistant variants
e
Flag
GAPDH
TRF1 shRNA
Vector
TRF1-WT
TRF1-2D
3Flag-TRF1 shRNA-resistant variants
f
24 hr colcemid
HT1080
HCT116
Metaphase-TIF/Cell (n)
p=0.3508
<0.0001
<0.0001
>0.9999
<0.0001
<0.0001
TRF1 shRNA
Vector
TRF1-WT
TRF1-T358A
3Flag-TRF1 shRNA-resistant variants
g
IMR90 E6E7 hTERT, 24 hr colcemid
Vector
mScarlet
mClover3
WT-TRF1
WT-TRF2
TRF1-3A
WT-TRF2
TRF1-2D
WT-TRF2
TRF2-2A
WT-TRF1
TRF2-2D
WT-TRF1
mScarlet
mClover3
Merge
Enlarged
h
HT1080 6TG
TRF1 shRNA
Flag-TRF1
Flag-TRF1-T358A
pTRF1 (T358)
Flag
pTRF1
TRF1
IgG
TRF1
Vinculin
2 mM Thy
150 ng mL-1 Noc
Alk. PPase (100 U)
Input
IP:Flag
Input
IP:Flag

Supplementary Figure 2. AURKB consensus sites at TRF1-S354 and -T358 participate in MAD telomere deprotection. a. Immunoblots of whole cell extracts from Control or TRF1 shRNA IMR90 E6E7 hTERT (representative example of n ≥ 3 biological replicates). **b.** Immunoblots of whole cell extracts from Control or TRF1 shRNA IMR90 E6E7 hTERT expressing the indicated shRNA resistant Flag-TRF1 alleles (representative of n ≥ 3 biological replicates). **c.** Metaphase-telomere deprotection induced foci (TIF) assays stained with γH2AX immunofluorescence (red), telomere FISH (TelC, green), and DAPI (blue) in control or TRF1 shRNA IMR90 E6E7 hTERT expressing the indicated shRNA-resistant FLAG-TRF1 alleles. All images are from cells treated with 100 ng mL^{-1} colcemid for 24 hours prior to sample collection (representative images from n ≥ 3 biological replicates). Scale bar, 10 μm. **d, e.** Immunoblots of whole cell extracts from control or TRF1 shRNA IMR90 E6E7 hTERT expressing the indicated shRNA resistant TRF1 alleles (representative of n = 3 biological replicates). **f.** Quantitation of metaphase-TIF in TRF1 shRNA HT1080 and TRF1 shRNA HCT116 cells expressing the indicated TRF1 alleles. Cells were treated with 24 hours of 100 ng mL^{-1} colcemid (n = 2 biological replicates; 30 metaphases per replicate for HT1080, 25 and 30 metaphases for HCT116, compiled into a Tukey boxplot, Kruskal-Wallis followed by Dunn's multiple comparisons test). **g.** Representative images of n = 2 biological replicates for mScarlet- or mClover-tagged TRF1 and TRF2 variants in cells treated with 100 ng mL^{-1} colcemid for 24 hours. TRF1-3A, S296A/S354A/T358A; TRF2-2A, S62A/S65A; TRF2-2D, S62D/S65D. Scale bar, 10 μm, DNA is stained with DAPI (blue). **h.** Immunoblots of Flag immuno-precipitates from TRF1 shRNA HT1080 6TG cells expressing shRNA-resistant WT Flag-TRF1 or Flag-TRF1-T358A. Where indicated, cells were synchronised with a double-thymidine block (Thy) and released in the presence or absence of 150 ng mL^{-1} nocodazole (Noc) for 16 hours before sample collection. Where indicated extracts were treated with alkaline phosphatase (Alk. PPase). The pTRF1-T358 band is indicated. Representative of n = 3 biological replicates. Quantitative source data are provided as a Source Data file and source blots at the end of this document.

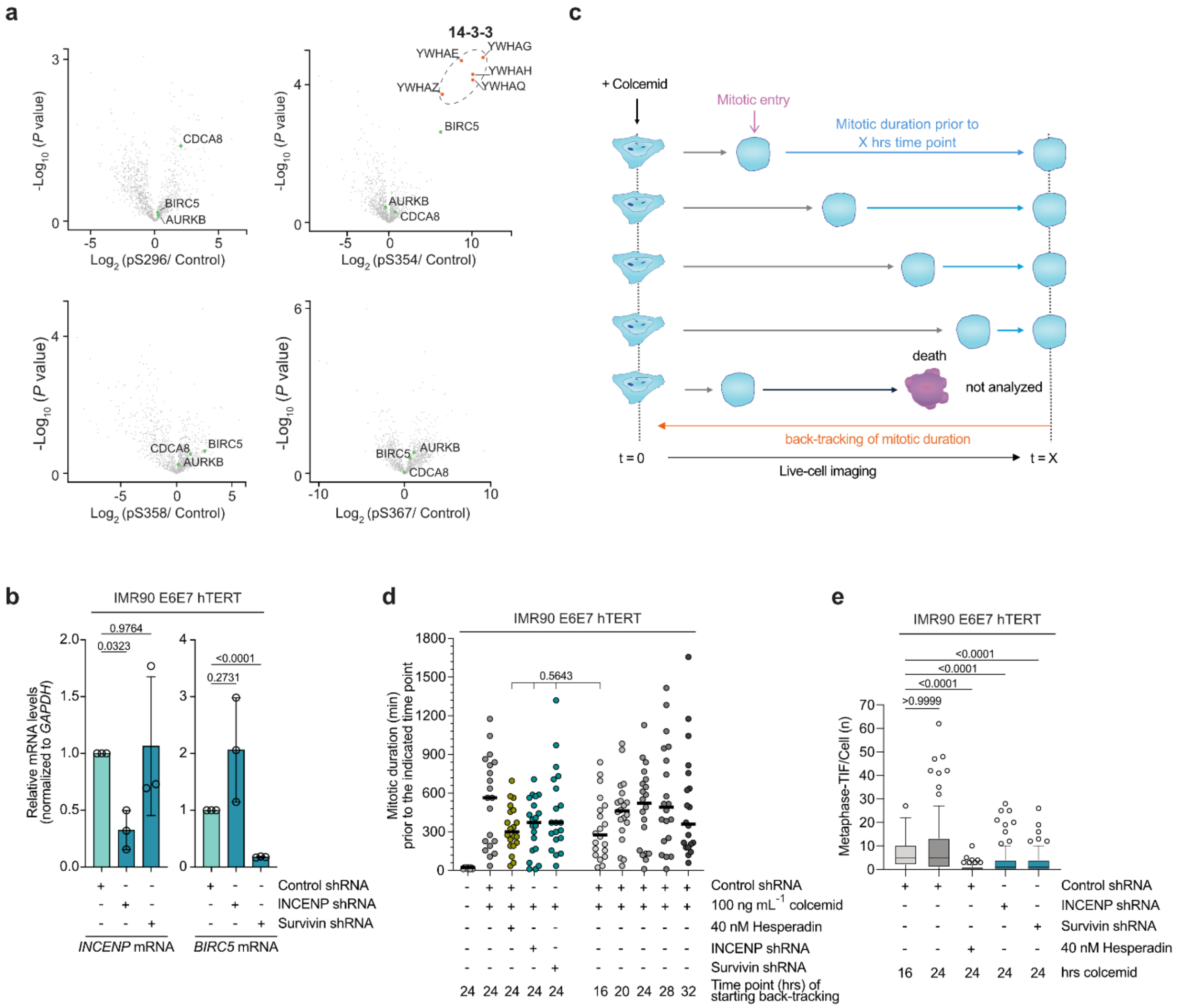

Supplementary Figure 3. Survivin interacts with TRF1 phospho-peptides and is required for MAD telomere deprotection. a. Volcano plots of LC-MS/MS analysis of TRF1 peptide pulldowns as depicted in Fig. 3a. LFQ values from proteins detected in phosphorylated TRF1 peptide pulldowns were compared to respective values in non-phosphorylated controls and plotted as a function of Log_2 fold-change and $-Log_{10}$ p-value. Enriched CPC members and 14-3-3 phospho-binding proteins are indicated (one-tailed student's T-test of n = 3 biological replicates). **b.** Gene expression in IMR90 E6E7 hTERT five days after shRNA transduction measured by RT-qPCR and normalized to *GAPDH* (mean +/- s.d., n = 3 biological replicates, RM one-way ANOVA with the Geisser-Greenhouse correction). **c.** Schematic of mitotic duration analysis in (**d**). Cells were exposed to 100 ng mL^{-1} colcemid at the beginning of the movie. Mitotic cells at the time point of interest were backtracked to determine mitotic duration. **d.** Mitotic duration before the indicated time point in colcemid-treated IMR-90 E6E7 hTERT cells expressing indicated shRNA or exposed to Hesperadin (median, n = 20 except for mock n = 10, Kruskal-Wallis test). **e.** Metaphase-telomere deprotection induced foci (TIF) in IMR90 E6E7 hTERT fibroblasts five days post-transduction with the indicated shRNAs. Cultures were treated with 100 ng mL^{-1} colcemid with or without 40 nM Hesperadin for indicated hours prior to sample collection (n = 3 biological replicates of 30 metaphases per replicate, compiled into a Tukey Box plot, Kruskal-Wallis followed by Dunn's multiple comparisons test). Source data are provided as a Source Data file.

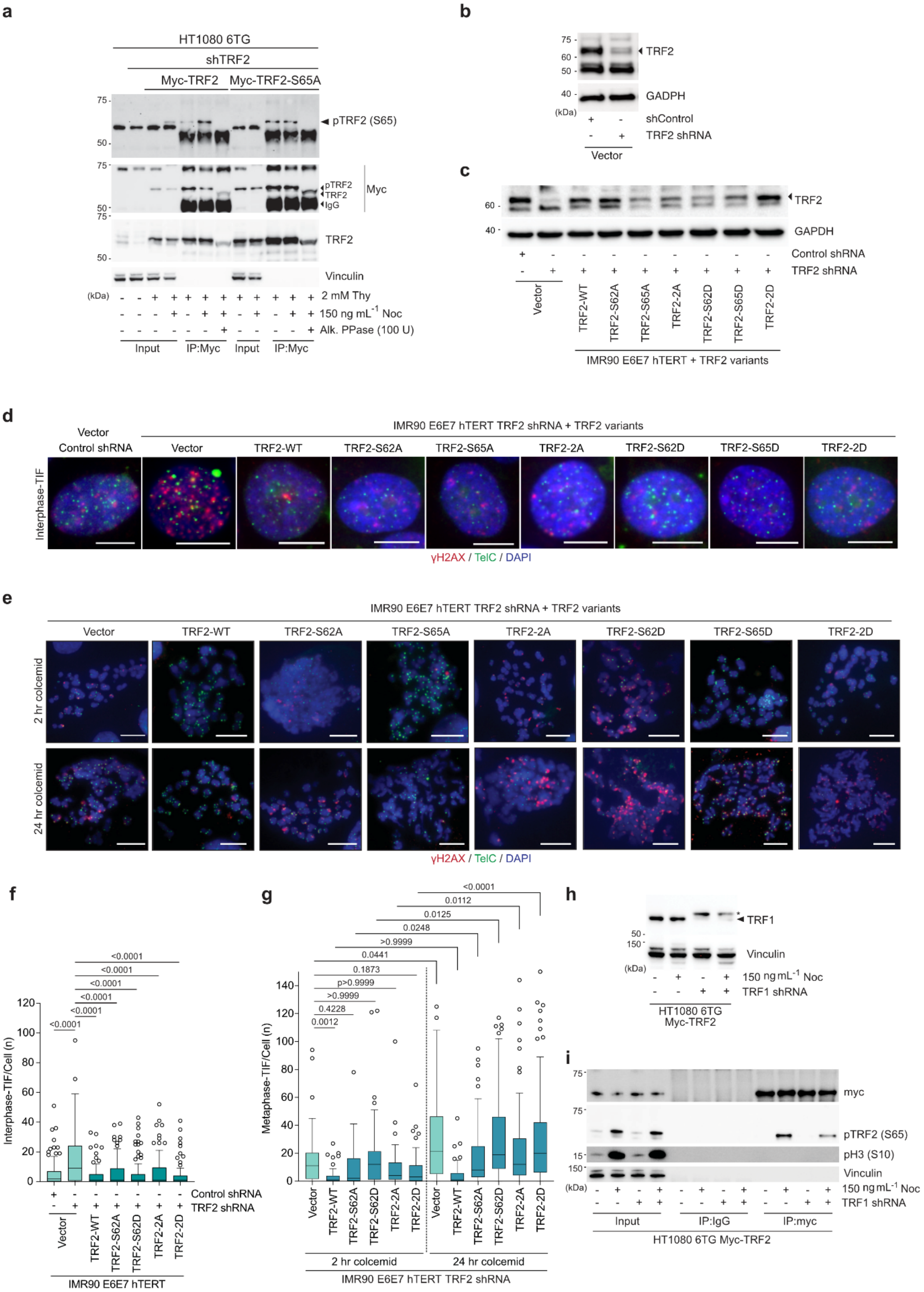
a
HT1080 6TG
shTRF2
Myc-TRF2
Myc-TRF2-S65A
pTRF2 (S65)
pTRF2
TRF2
IgG
Myc
TRF2
Vinculin
(kDa)
2 mM Thy
150 ng mL-1 Noc
Alk. PPase (100 U)
Input
IP:Myc
b
TRF2
GADPH
shControl
TRF2 shRNA
Vector
c
TRF2
GAPDH
Control shRNA
TRF2 shRNA
Vector
TRF2-WT
TRF2-S62A
TRF2-S65A
TRF2-2A
TRF2-S62D
TRF2-S65D
TRF2-2D
IMR90 E6E7 hTERT + TRF2 variants
d
IMR90 E6E7 hTERT TRF2 shRNA + TRF2 variants
Vector
Control shRNA
Interphase-TIF
γH2AX / TelC / DAPI
e
IMR90 E6E7 hTERT TRF2 shRNA + TRF2 variants
2 hr colcemid
24 hr colcemid
γH2AX / TelC / DAPI
f
Interphase-TIF/Cell (n)
Control shRNA
TRF2 shRNA
IMR90 E6E7 hTERT
g
Metaphase-TIF/Cell (n)
2 hr colcemid
24 hr colcemid
IMR90 E6E7 hTERT TRF2 shRNA
h
TRF1
Vinculin
150 ng mL-1 Noc
TRF1 shRNA
HT1080 6TG
Myc-TRF2
i
myc
pTRF2 (S65)
pH3 (S10)
Vinculin
150 ng mL-1 Noc
TRF1 shRNA
Input
IP:IgG
IP:myc
HT1080 6TG Myc-TRF2

Supplementary Figure 4. The TRF2 basic domain functions in mitosis-specific telomere protection. a. Immunoblots of Myc immuno-precipitates from TRF2 shRNA HT1080 6TG expressing WT Myc-TRF2 or Myc-TRF2-S65A. Where indicated, cells were double-thymidine block (Thy) synchronised, released in the presence or absence of 150 ng mL^{-1} Nocodazole (Noc) for 16 hours, and/or treated with alkaline phosphatase (Alk. PPase). Representative of n = 3 biological replicates. **b, c.** Western blots of whole cell extracts from IMR90 E6E7 hTERT TRF2 transduced with Control or TRF2 shRNA (b) and the indicated TRF2 alleles (c) (representative of n ≥ 3 biological replicates). **d, e.** Examples of Interphase-telomere deprotection induced foci (TIF) (d) and Metaphase-TIF (e) stained with γH2AX immunofluorescence (red), telomere FISH (TelC, green), and DAPI (blue) from IMR90 E6E7 hTERT transduced with the indicated shRNA and TRF2 alleles (representative of n ≥ 3 biological replicates). In (e) cultures were treated with 100 ng mL^{-1} colcemid for 2 or 24 hours prior to sample collection. **f, g.** Quantitation of interphase-TIF (f) and metaphase-TIF (g) in TRF2 shRNA IMR90 E6E7 hTERT expressing TRF2-WT or the indicated TRF2 variants. For (f), n = 3 biological replicates of 45 nuclei per replicate, compiled into a Tukey boxplot, Kruskal-Wallis followed by Dunn's multiple comparisons test. For (g), cells were treated with 2 or 24 hours of 100 ng mL^{-1} colcemid (n = 3 biological replicates of 15 and 30 metaphases per replicate for 2 hours and 24 hours colcemid, respectively, compiled into a Tukey boxplot, Kruskal-Wallis followed by Dunn's multiple comparisons test). Data for Vector and TRF2-WT are the same as in Fig. 4d and 4e. **h**, **i**. Immunoblots of cell lysates (h) or anti-Myc immuno-precipitates (i) from TRF1 shRNA HT1080 6TG cells expressing Myc-TRF2-WT. Cells were thymidine block synchronised, released, and treated with 150 ng mL^{-1} of nocodazole for 16 hours. Representative of n = 2 experimental replicates. Quantitative source data are provided as a Source Data file and source blots at the end of this document.

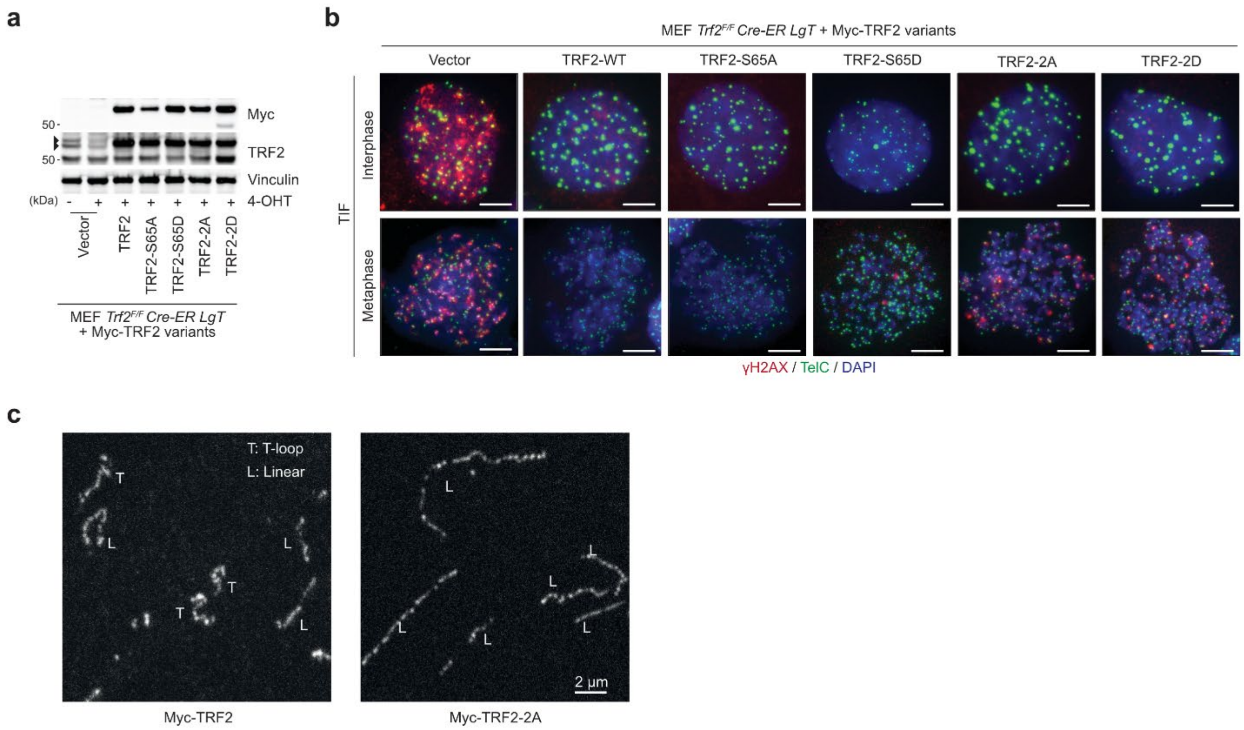

Supplementary Figure 5. Imaging telomere deprotection and telomere macromolecular structure in *Trf2*$^{F/F}$ *Cre-ER LgT* MEFs. a. Immunoblots of whole cell extracts derived from *Trf2*$^{F/F}$ *Cre-ER LgT* MEFs expressing the indicated Myc-tagged hTRF2 alleles. Where indicated the cultures were treated with 4-Hydroxytamoxifen (4-OHT) for 36 hours prior to sample collection to delete endogenous *mTrf2* (n = 1). **b.** Representative interphase-telomere deprotection induced foci (TIF) and metaphase-TIF images from *Trf2*$^{F/F}$ *Cre-ER LgT* MEFs expressing hTRF2-WT or the indicated hTRF2 variants. In all conditions endogenous *mTrf2* was deleted by 4-OHT addition 36 hours prior to sample fixation. Samples were stained with combined γH2AX immunofluorescence (red) and telomere FISH (TelC, green). The DNA is labelled with DAPI (blue). For Metaphase-TIF assays, cells were treated with 400 ng mL^{-1} Nocodazole for 14 hours prior to sample collection. Scale bar, 10 µm. Representative from n = 3 biological replicates. **c.** Representative fields from AiryScan microscopy of telomere macromolecular structure. *Trf2*$^{F/F}$ *Cre-ER LgT* MEFs expressing Myc-TRF2-WT or Myc-TRF2-2A (S62A and S65A) were treated with 4-OHT for 36 hours prior to sample collection to delete endogenous *mTrf2*. Cultures were treated with 400 ng mL^{-1} Nocodazole for 14 hours and collected by mitotic shake-off before trioxsalen cross-linked in situ, chromatin spreading on coverslips through cytocentrifugation, and telomere FISH staining. Linear (L) and t-loops (T) are shown. Scale bar, 2 µm. Representative of n = 3 biological replicates. Source blots are provided at the end of this document.

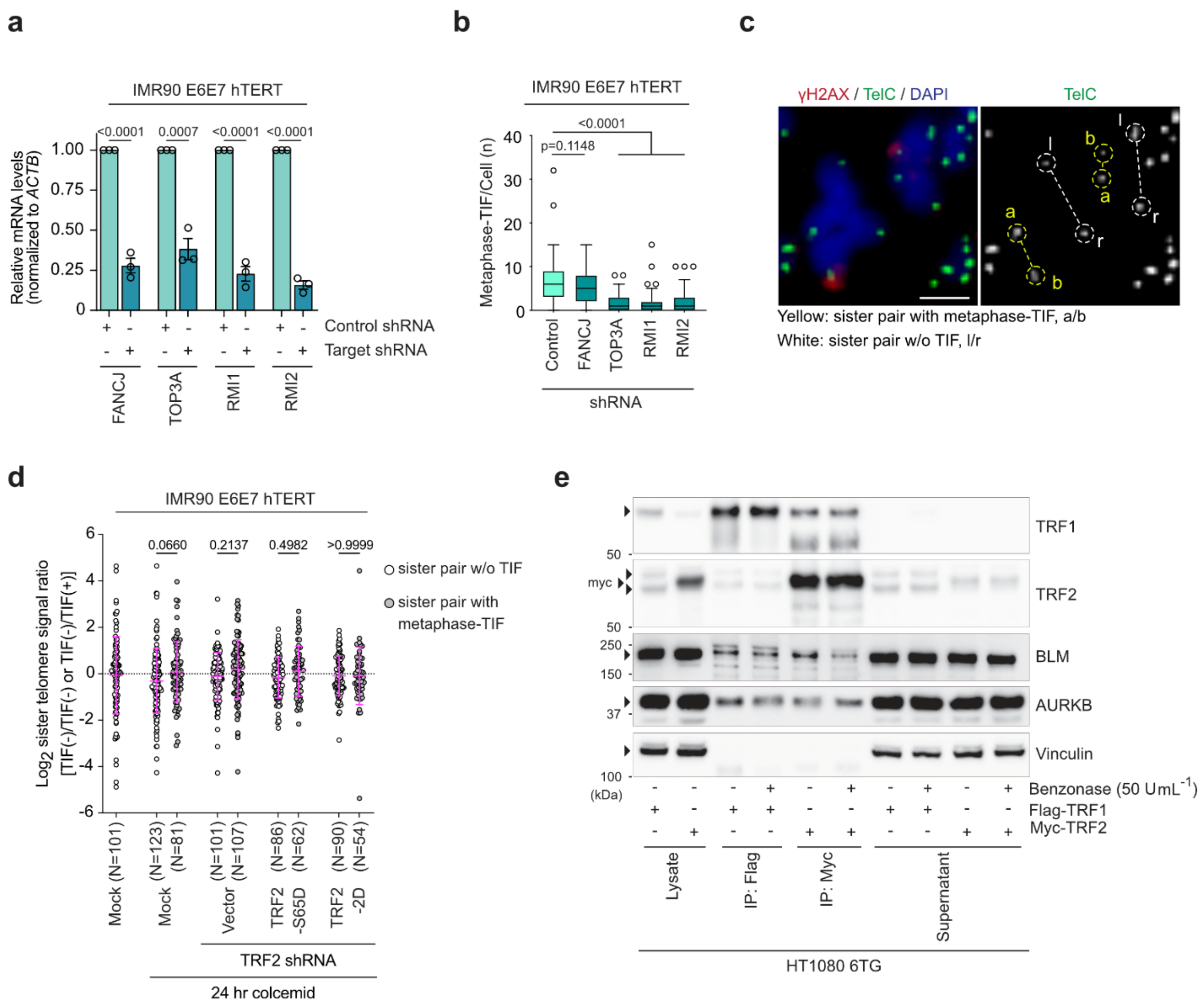

a
IMR90 E6E7 hTERT
Relative mRNA levels (normalized to ACTB)
<0.0001
0.0007
<0.0001
<0.0001
Control shRNA
Target shRNA
FANCJ
TOP3A
RMI1
RMI2
b
IMR90 E6E7 hTERT
Metaphase-TIF/Cell (n)
<0.0001
p=0.1148
Control
FANCJ
TOP3A
RMI1
RMI2
shRNA
c
γH2AX / TelC / DAPI
TelC
Yellow: sister pair with metaphase-TIF, a/b
White: sister pair w/o TIF, l/r
d
IMR90 E6E7 hTERT
Log2 sister telomere signal ratio [TIF(-)/TIF(-) or TIF(-)/TIF(+)]
0.0660
0.2137
0.4982
>0.9999
sister pair w/o TIF
sister pair with metaphase-TIF
Mock (N=101)
Mock (N=123) (N=81)
Vector (N=101) (N=107)
TRF2 -S65D (N=86) (N=62)
TRF2 -2D (N=90) (N=54)
TRF2 shRNA
24 hr colcemid
e
TRF1
TRF2
BLM
AURKB
Vinculin
myc
(kDa)
Benzonase (50 UmL-1)
Flag-TRF1
Myc-TRF2
Lysate
IP: Flag
IP: Myc
Supernatant
HT1080 6TG

Supplementary Figure 6. BTR promotes MAD telomere deprotection without telomere shortening. a. Relative gene expression in shRNA transduced IMR90 E6E7 hTERT. For each targeted gene, transcription was measured by RT-qPCR and normalized to *ACTB* (mean +/- s.e.m., n = 3 biological replicates, unpaired two-tailed t test). **b**. Metaphase-telomere deprotection induced foci (TIF) in IMR90 E6E7 hTERT fibroblasts five days post-transduction with the indicated shRNAs. Cultures were treated with 100 ng mL^{-1} colcemid for 24 hours prior to sample collection (n = 3 biological replicates of 30 metaphases per replicate, compiled into a Tukey Box plot, Kruskal-Wallis followed by Dunn's multiple comparisons test). **c**. Measurement of inter-chromatid telomere length variability. On metaphase-TIF assays stained with γH2AX IF (red), telomere FISH (TelC, Green), and DAPI, we identified sister telomeres without γH2AX staining (sister pair without (w/o) metaphase-TIF), or where a single chromatid was γH2AX positive (sister pair with metaphase-TIF). Telomere length for each chromatid was measured by FISH intensity. We calculated the ratio between sister telomere lengths and plotted as $\log_2$ values in (d). **d**. Ratio of sister telomere lengths as described in (c) for mock or TRF2 shRNA transduced IMR90 E6E7 hTERT ± the indicated exogenous TRF2 alleles. Where indicated, cultures were treated with 100 ng mL^{-1} colcemid for 24 hours prior to sample collection (mean ± s.d., N pairs, ordinary one-way ANOVA followed by Šídák's multiple comparisons test, F = 2.048, DF = (7, 696)). **e.** Immunoblots of anti-Flag and anti-Myc immuno-precipitates from HT1080 6TG cells expressing TRF1 shRNA with shRNA-resistant Flag-TRF1-WT and TRF2 shRNA with Myc-TRF2-WT, respectively. Cells were synchronised with a thymidine block, released, and treated with 150 ng $mL^{-1}$of nocodazole for 16 hours. Following sample collection and extract preparation, the indicated lysates were incubated with 50 U mL^{-1} Benzonase for 15 min on ice prior to immunoprecipitation. Representative of n = 2 biological replicates. Quantitative source data are provided as a Source Data file and source blots at the end of this document.

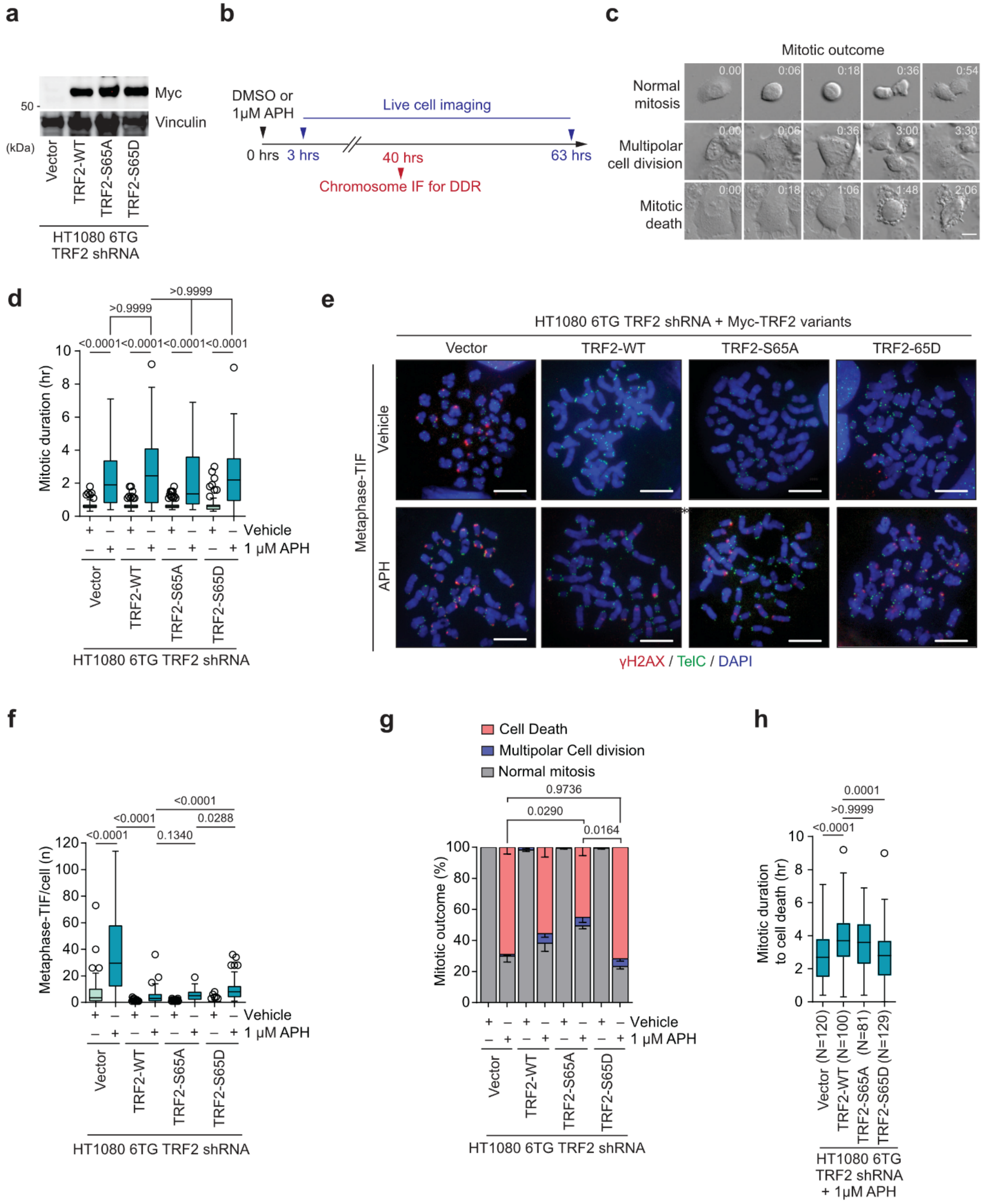
a
Myc
Vinculin
50
(kDa)
Vector
TRF2-WT
TRF2-S65A
TRF2-S65D
HT1080 6TG TRF2 shRNA
b
DMSO or 1μM APH
Live cell imaging
0 hrs
3 hrs
40 hrs
63 hrs
Chromosome IF for DDR
c
Mitotic outcome
Normal mitosis
Multipolar cell division
Mitotic death
d
Mitotic duration (hr)
Vehicle
1 μM APH
HT1080 6TG TRF2 shRNA
e
HT1080 6TG TRF2 shRNA + Myc-TRF2 variants
Vector
TRF2-WT
TRF2-S65A
TRF2-65D
Metaphase-TIF
Vehicle
APH
γH2AX / TelC / DAPI
f
Metaphase-TIF/cell (n)
Vehicle
1 μM APH
HT1080 6TG TRF2 shRNA
g
Cell Death
Multipolar Cell division
Normal mitosis
Mitotic outcome (%)
Vehicle
1 μM APH
HT1080 6TG TRF2 shRNA
h
Mitotic duration to cell death (hr)
Vector (N=120)
TRF2-WT (N=100)
TRF2-S65A (N=81)
TRF2-S65D (N=129)
HT1080 6TG TRF2 shRNA + 1μM APH

Supplementary Figure 7. TRF2 phosphorylation promotes mitotic death after lethal replication stress. a. Immunoblots of whole cell extracts derived from HT1080 6TG shTRF2 cells expressing the indicated Myc-TRF2 alleles (representative of n = 3 biological replicates). **b.** Timeline of the experimentation in panels (c-h). **c.** Captures from differential interference contrast live imaging of HT1080 6TG cells treated with 1 µM Aphidicolin (APH). Mitotic outcomes are indicated. Time is shown as hours: minutes relative to the first image. Scale bar, 20 µm. Representative of n = 3 biological replicates. **d.** Mitotic duration for TRF2 shRNA HT1080 6TG cells expressing the indicated Myc-TRF2 alleles. Cells were observed for 60 hours in cultures treated with or without 1 µM Aphidicolin (APH) (mean +/- s.e.m., n = 3 biological replicates of 60 mitotic cells per replicate, Kruskal-Wallis followed by Dunn's multiple comparisons test). **e.** Representative metaphase-telomere deprotection induced foci (TIF) images stained with γH2AX immunofluorescence (red), telomere FISH (TelC, green), and DAPI (blue) from TRF2 shRNA HT1080 6TG cultures expressing the indicated Myc-TRF2 alleles. Cultures were treated with 1 µM Aphidicolin (APH) for 40 hours before sample collection. Scale bar, 10 µm. Representative of n = 3 biological replicates. **f**. Quantitation of metaphase-TIF from TRF2 shRNA HT1080 6TG cultures expressing the indicated Myc-TRF2 alleles. Where indicated, cultures were treated with 1 µM Aphidicolin (APH) for 40 hours before sample collection (n = 3 biological replicates of 30 metaphases per replicate compiled into a Tukey Box Plot, Kruskal-Wallis followed by Dunn's multiple comparisons test). **g.** Mitotic outcomes from the experiment in (d) (mean +/- s.e.m., % mitotic outcome from n = 3 biological replicates, Ordinary one-way ANOVA followed by Tukey's multiple comparisons test for % Cell Death). **h.** Mitotic duration until death from the experiment in (d) (mean +/- s.e.m., N mitotic cells from n = 3 biological replicates, Kruskal-Wallis followed by Dunn's multiple comparisons test). Quantitative Source data are provided as a Source Data file and source blots at the end of this document.

Source blots for the Supplementary Data

Supplementary Fig. 1b

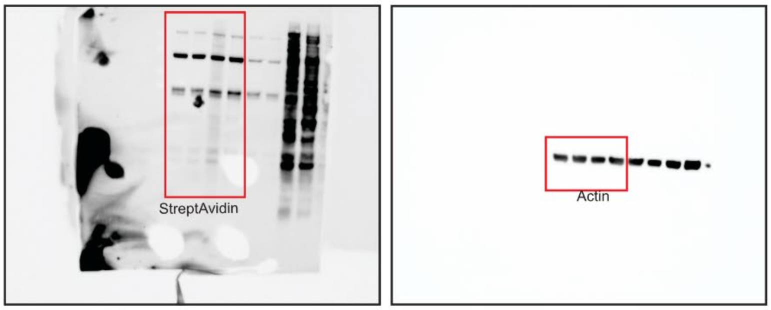

Supplementary Fig. 2a

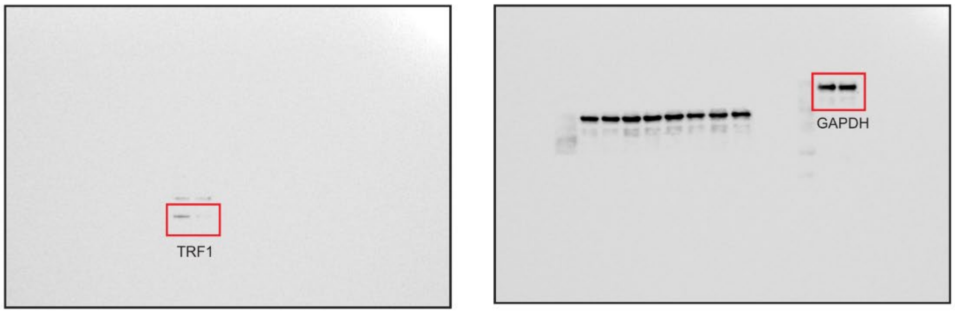

Supplementary Fig. 2b

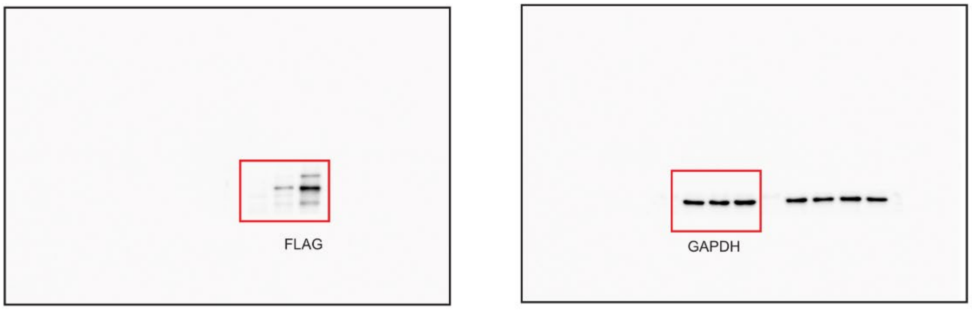

Supplementary Fig. 2d

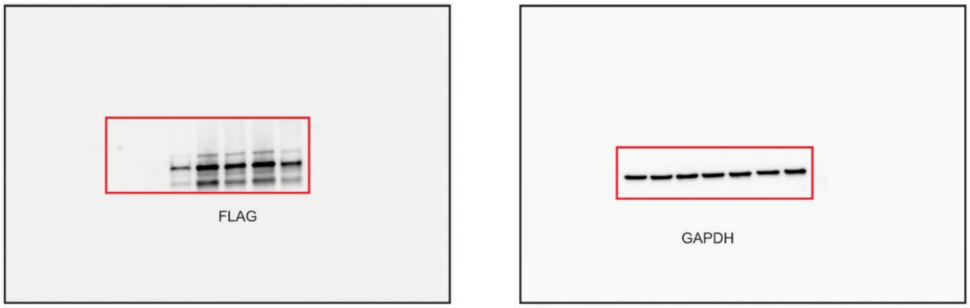

Supplementary Fig. 2e

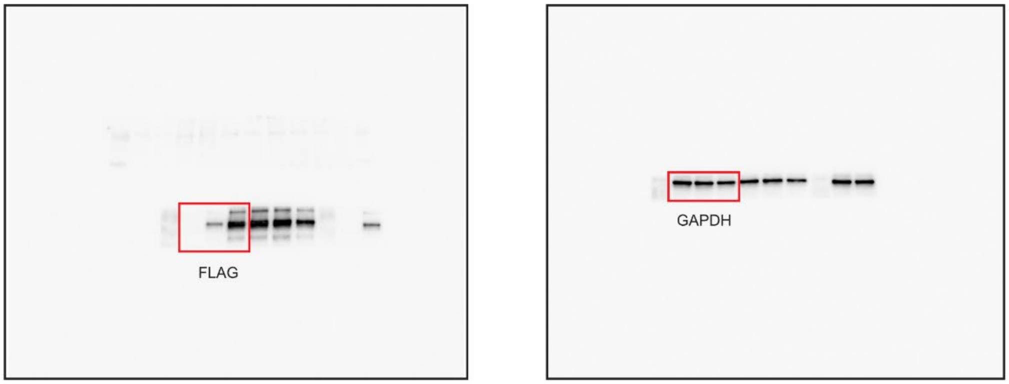

Supplementary Fig. 2h

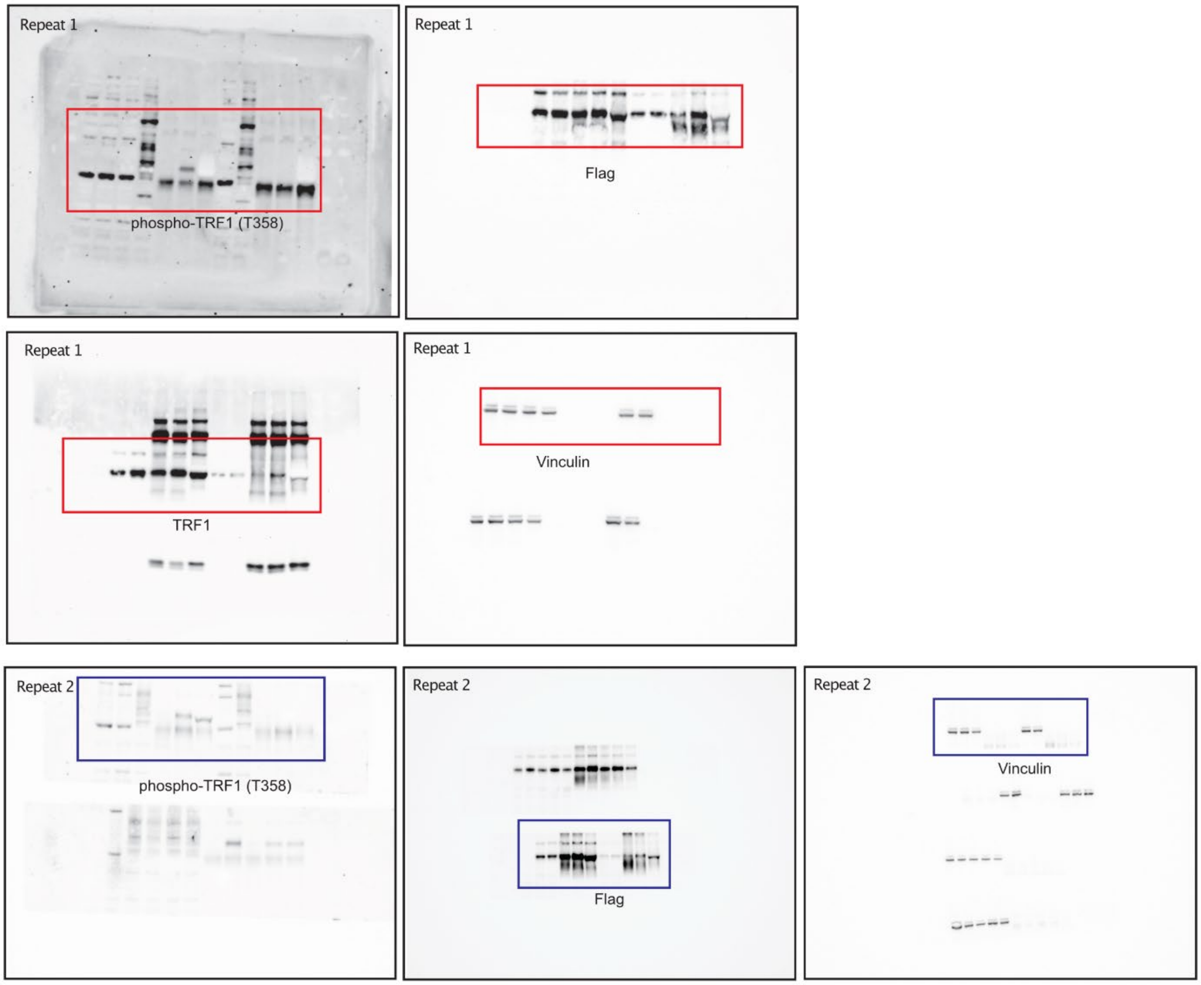

Supplementary Fig. 4a

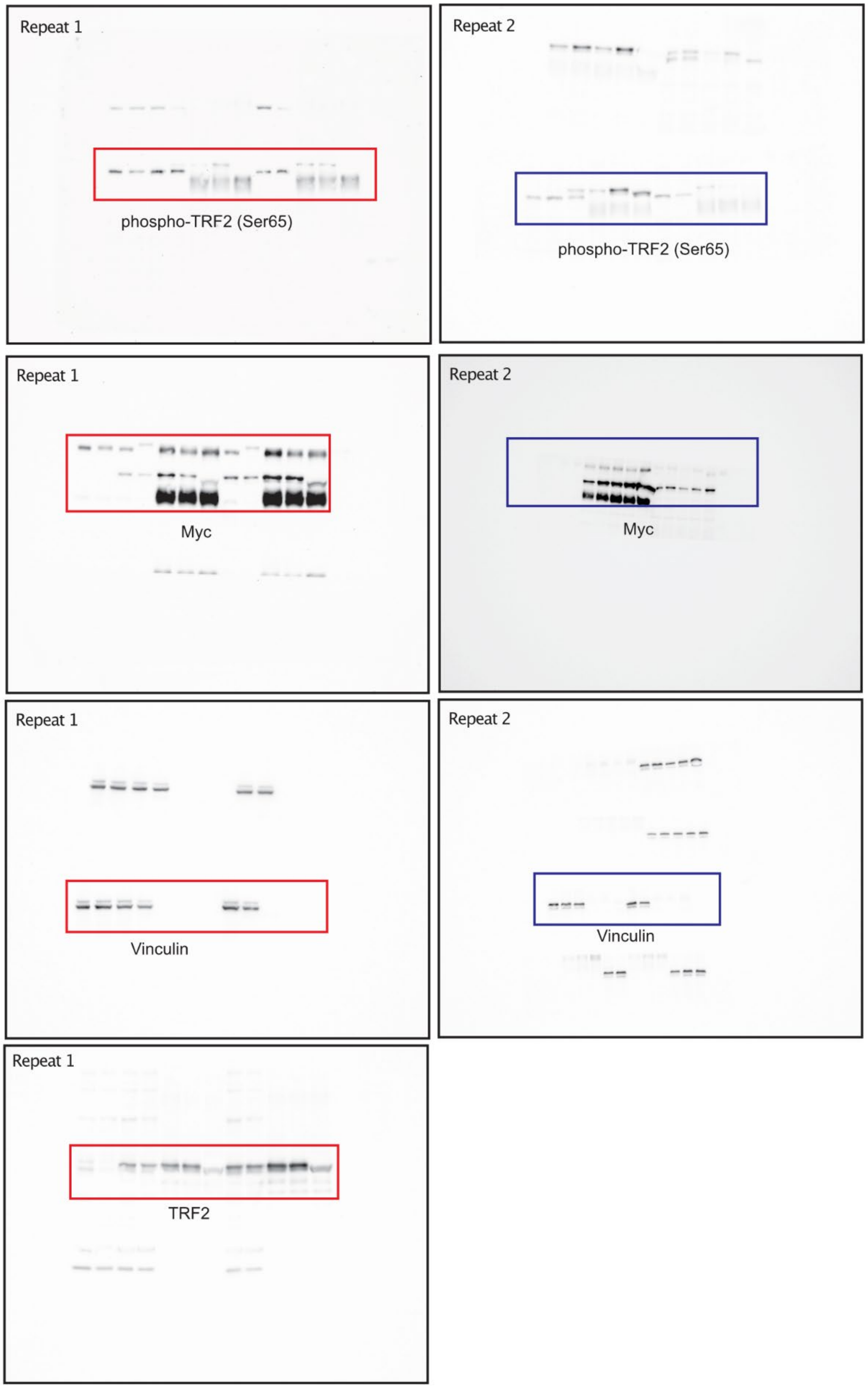

Supplementary Fig. 4b

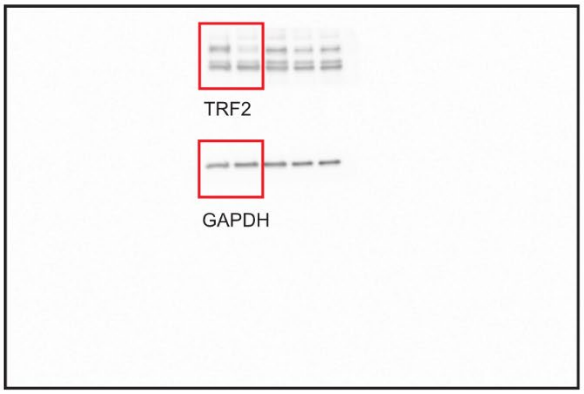

Supplementary Fig. 4c

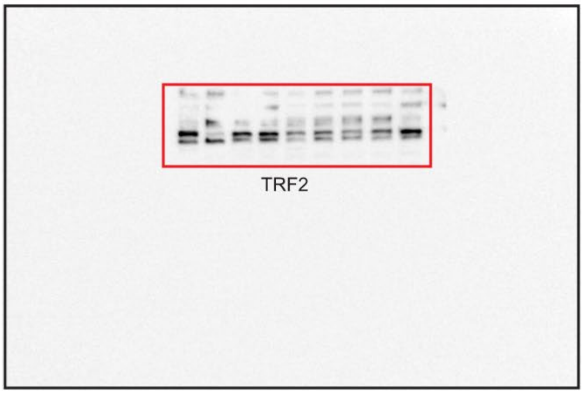

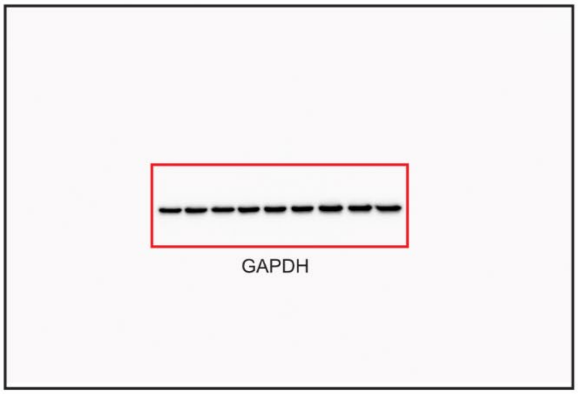

Supplementary Fig. 4h

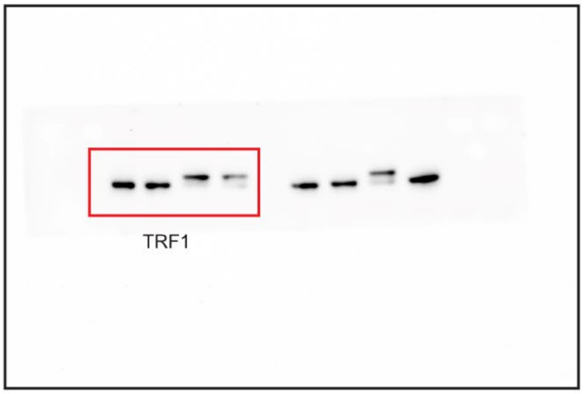

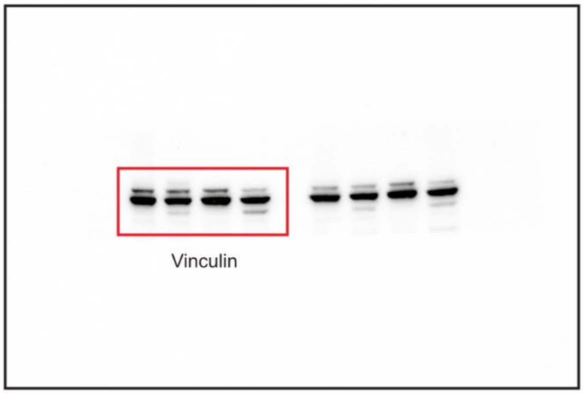

Supplementary Fig. 4i

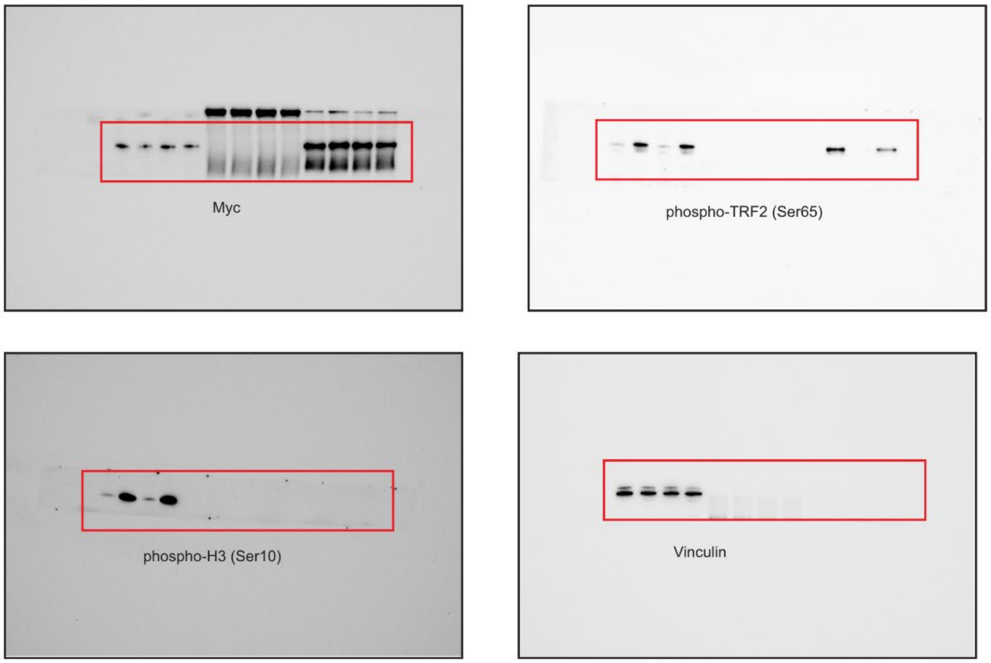

Supplementary Fig. 5a

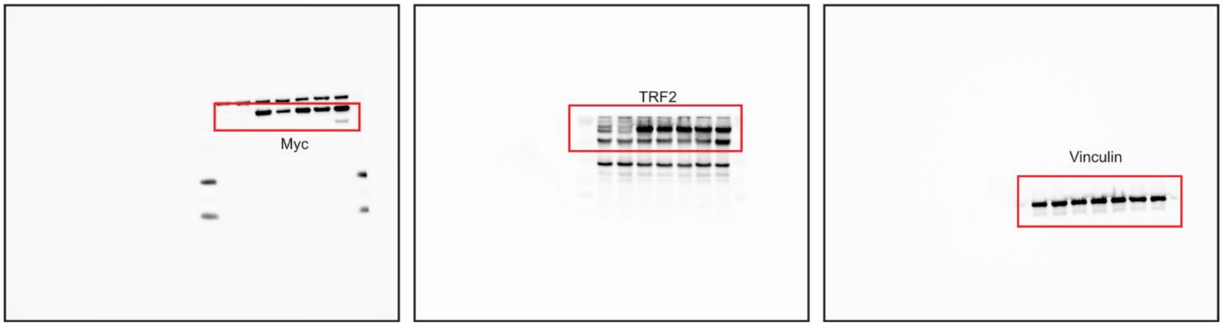

Supplementary Fig. 6e

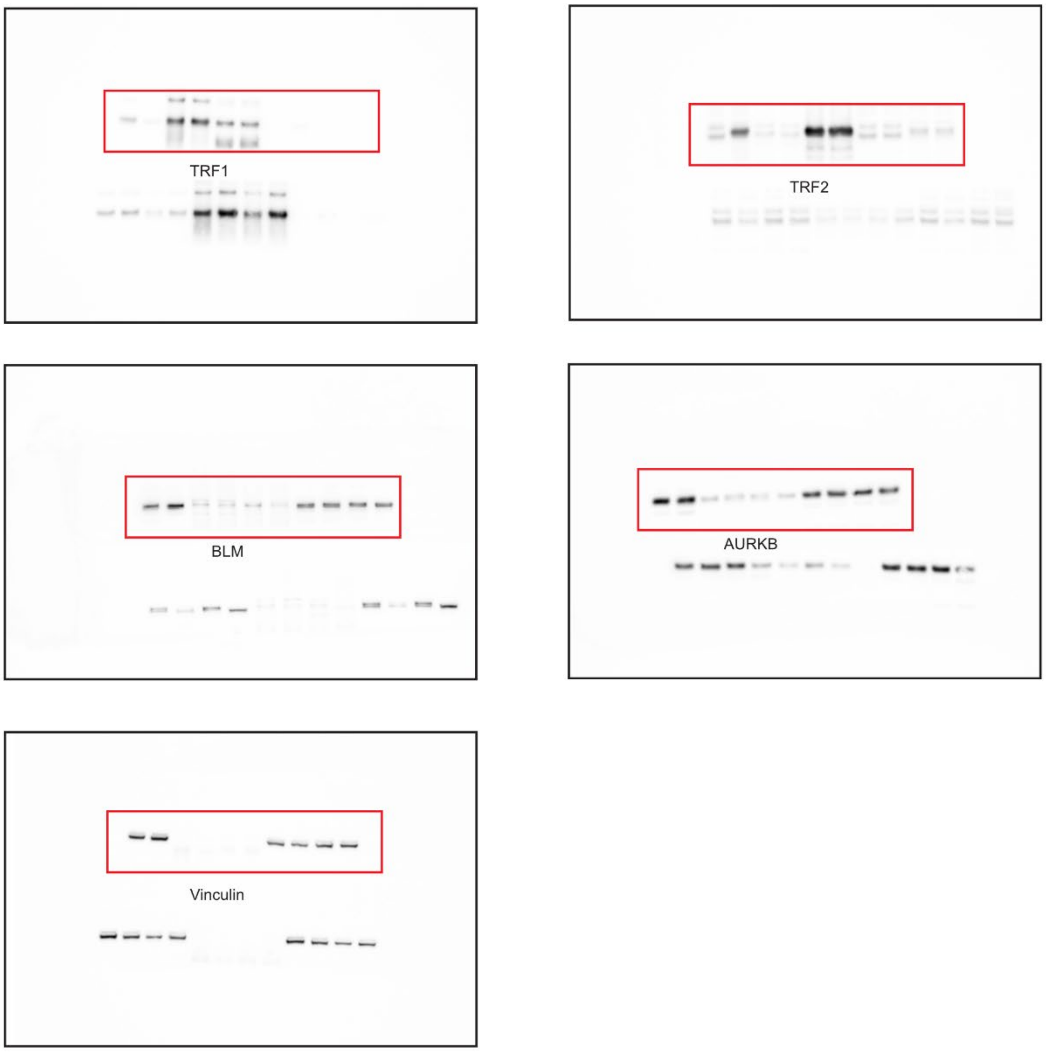

Supplementary Fig. 7a

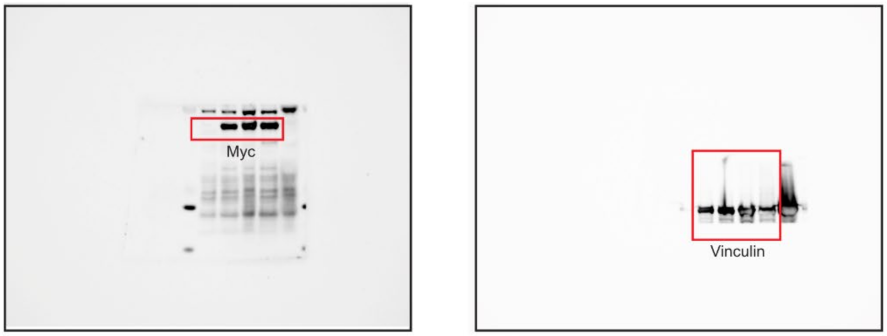

Supplement: Supplementary file 1 — Supplementary Information [file 41467_2025_57456_MOESM1_ESM.pdf]
