# Supplementary material for: A CPC-shelterin-BTR axis regulates mitotic telomere deprotection
Source: Nat Commun. 2025 Mar 17;16:2277. doi: 10.1038/s41467-025-57456-8 (PMC11914695; doi:10.1038/s41467-025-57456-8)
Supplement: Supplementary file 2 — Description of Additional Supplementary Files [file 41467_2025_57456_MOESM2_ESM.pdf]

Description of Additional Supplementary Files

File Name: Supplementary Data 1
Description: Plasmids used in this study.

File Name: Supplementary Data 2
Description: Primers used in this study.
