# Supplementary material for: A CPC-shelterin-BTR axis regulates mitotic telomere deprotection
Source: Nat Commun. 2025 Mar 17;16:2277. doi: 10.1038/s41467-025-57456-8 (PMC11914695; doi:10.1038/s41467-025-57456-8)
Supplement: Supplementary file 5 — Reporting Summary [file 41467_2025_57456_MOESM5_ESM.pdf]

nature portfolio

Corresponding author(s): Anthony Cesare and Makoto Hayashi

Last updated by author(s): Dec 19, 2024

Reporting Summary

Nature Portfolio wishes to improve the reproducibility of the work that we publish. This form provides structure for consistency and transparency in reporting. For further information on Nature Portfolio policies, see our Editorial Policies and the Editorial Policy Checklist.

Statistics

For all statistical analyses, confirm that the following items are present in the figure legend, table legend, main text, or Methods section.

n/a	Confirmed	
☐	☒	The exact sample size (*n*) for each experimental group/condition, given as a discrete number and unit of measurement
☐	☒	A statement on whether measurements were taken from distinct samples or whether the same sample was measured repeatedly
☐	☒	The statistical test(s) used AND whether they are one- or two-sided<br>*Only common tests should be described solely by name; describe more complex techniques in the Methods section.*
☒	☐	A description of all covariates tested
☒	☐	A description of any assumptions or corrections, such as tests of normality and adjustment for multiple comparisons
☐	☒	A full description of the statistical parameters including central tendency (e.g. means) or other basic estimates (e.g. regression coefficient) AND variation (e.g. standard deviation) or associated estimates of uncertainty (e.g. confidence intervals)
☐	☒	For null hypothesis testing, the test statistic (e.g. *F*, *t*, *r*) with confidence intervals, effect sizes, degrees of freedom and *P* value noted<br>*Give P values as exact values whenever suitable.*
☒	☐	For Bayesian analysis, information on the choice of priors and Markov chain Monte Carlo settings
☒	☐	For hierarchical and complex designs, identification of the appropriate level for tests and full reporting of outcomes
☒	☐	Estimates of effect sizes (e.g. Cohen's *d*, Pearson's *r*), indicating how they were calculated

Our web collection on statistics for biologists contains articles on many of the points above.

Software and code

Policy information about availability of computer code

Data collection	Mass spectrometry was performed within the CMRI Biomedical Proteomics Core facility using MS acquisition software is Xcalibur 4.2.47 (Thermo Fisher Scientific).<br>BIO-RAD Image Lab Touch v2.0.0.27<br>ZEISS Zen Blue, v2.0, 2.6<br>ZEISS Zen Black v2.3<br>Metasystems Metafer v4

Data analysis	MaxQaunt v1.6.0.16<br>Perseus v1.6.10.43<br>Excel (Office 356)<br>Prism 9.1.0 (221) and 10.0.0 (131)<br>Enrichr (Chen EY et al, BMC Bioinformatics 2013)<br>Uniprot Human Proteome – August 2018<br>BZ-X Analyzer 1.3.1.1<br>Zen Blue Desk v2.6<br>MetaSystems ISIS v5.5.8<br>Fiji/ImageJ<br>Photoshop 23.1.0<br>Illustrator 27.0.1 (64-bit)<br>Affinity Designer 1.10.6<br>SnapGene 3.0.3 and 5.0.8

For manuscripts utilizing custom algorithms or software that are central to the research but not yet described in published literature, software must be made available to editors and reviewers. We strongly encourage code deposition in a community repository (e.g. GitHub). See the Nature Portfolio guidelines for submitting code & software for further information.

Data

Policy information about availability of data

All manuscripts must include a data availability statement. This statement should provide the following information, where applicable:

- Accession codes, unique identifiers, or web links for publicly available datasets
- A description of any restrictions on data availability
- For clinical datasets or third party data, please ensure that the statement adheres to our policy

The mass spectrometry outputs, and MaxQuant analyses in this paper are available on the PRIDE database (PXD043281). The remaining datasets generated and/or analysed during the current study are available from M.T.H. and A.J.C. upon reasonable request.

Research involving human participants, their data, or biological material

Policy information about studies with human participants or human data. See also policy information about sex, gender (identity/presentation), and sexual orientation and race, ethnicity and racism.

Reporting on sex and gender	N/A
Reporting on race, ethnicity, or other socially relevant groupings	N/A
Population characteristics	N/A
Recruitment	N/A
Ethics oversight	N/A

Note that full information on the approval of the study protocol must also be provided in the manuscript.

Field-specific reporting

Please select the one below that is the best fit for your research. If you are not sure, read the appropriate sections before making your selection.

☒ Life sciences ☐ Behavioural & social sciences ☐ Ecological, evolutionary & environmental sciences

For a reference copy of the document with all sections, see nature.com/documents/nr-reporting-summary-flat.pdf

Life sciences study design

All studies must disclose on these points even when the disclosure is negative.

Sample size	No statistical method was applied to predetermine sample size. Sample sizes are consistent with published studies using similar experimentation (Van Ly et al, Molecular Cell 2018; Sarek et al Nature 2019; Ruis et al Nature 2021) and are reported in the figure legends. Five independent repeats of the interactomics were performed for each experimental setting. All other experiments were performed with two or more independent replicates based on standard field practice.
Data exclusions	No data exclusions.
Replication	Five independent repeats of the interactomics mass spectrometry were performed. All other experiments were performed on two or more independent replicates as described in the figure legends.

Randomization	For metaphase-TIF analysis in IMR90 E6E7 hTERT fibroblasts, images were taken blindly by lab technicians and quantified using automated BZ-X Analyzer software. For metaphase-TIF analysis in all other cell lines images were captured automatically using Metafer. For the analysis of telomere macromolecular structure and live imaging, portions of the slide or culture dish to image were chosen at random.
Blinding	IMR90 E6E7 hTERT metaphase-TIF analysis the data were quantified using automated BZ-X Analyzer software. For analysis of metaphase-TIF in other cells lines, and macromolecular telomere structure, researchers were blinded to experimental conditions. For live imaging and interphase-TIF analysis researchers were not blinded due to impracticality.

Reporting for specific materials, systems and methods

We require information from authors about some types of materials, experimental systems and methods used in many studies. Here, indicate whether each material, system or method listed is relevant to your study. If you are not sure if a list item applies to your research, read the appropriate section before selecting a response.

Materials & experimental systems

n/a	Involved in the study
☐	☒ Antibodies
☐	☒ Eukaryotic cell lines
☒	☐ Palaeontology and archaeology
☒	☐ Animals and other organisms
☒	☐ Clinical data
☒	☐ Dual use research of concern
☒	☐ Plants

Methods

n/a	Involved in the study
☒	☐ ChIP-seq
☒	☐ Flow cytometry
☒	☐ MRI-based neuroimaging

Antibodies

Antibodies used

Antibodies and their dilution for Western blotting were as follows: TRF2 (NB110-57130SS, Novus Biologicals), 1:1000; TRF1 (sc-56807, SantaCruz), 1:1000; TRF1 rabbit polyclonal antibody (Ishikawa lab, used in Supplementary Figure S2), 1:1,000; BLM (NB100-214, NovusBio), 1:5000; Flag (F1804, Sigma), 1:2000; Myc (9B11 Cell Signalling Technology), 1:1000; Top3A (14525-1-AP, Proteintech), 0.3 µg mL-1; StreptAvidin-HRP (S911, ThermoFisher), 1:1000; INCENP (ab-12183, AbCam), 1:2000; Aurora B (ab-2254, AbCam), 1:1000; Borealin (ab74473, Abcam), 1:1000; Survivin (NB500-201, Novus Biologicals), 1:1000; beta-Actin (A5441, Sigma), 1:20,000; Actin (MAB1501R, Millipore), 1:10,000; Vinculin (V9131, Sigma), 1:5000; GAPDH (MAB374, Millipore), 1:5000; GAPDH (M171-3, MBL), 1:1000; Goat anti-mouse HRP (P044701, DAKO Agilent), 1:5000; and Goat anti-Rabbit HRP (P044801, DAKO Agilent). For immunofluorescence we used: γ-H2AX (Clone 2F3, Biolegend) 1:200 or (05-636, Merck Millipore), 1:500; Alexa-568 goat anti-mouse (A11031, Invitrogen); 1:10,000; and Streptavidin Alexa-488 (S32354, Invitrogen), 1:1,000.

Polyclonal rabbit antibodies against phopsho-TRF1 and TRF2 were generated by Genscript. Briefly, animals were immunized with peptides corresponding to pTRF1-T358 (SRRA(p)TESRIPVSKS), and pTRF2-Ser65 (ASRS(p)SGRARRGRHEC). Antibodies were purified by antigen affinity purification selecting for minimized cross adsorption and validated using indirect ELISA. For western blotting, antibodies were diluted in 5% BSA / TBS-T at a concentration of 0.2 µg mL-1.

Validation

1. Antibodies validated in this study using siRNA depletion: TRF2 (NB110-57130SS, Novus Biologicals), 1:1000; TRF1 (sc-56807, SantaCruz), 1:1000; TRF1 rabbit polyclonal antibody (Ishikawa lab, used in Extended data Figure 3) , 1:1,000; BLM (NB100-214, NovusBio), 1:2000; Top3A (14525-1-AP, Proteintech).

2. Antibodies validated in this study by over-expression: Flag (F1804, Sigma), 1:2000; Myc (9B11 Cell Signalling Technology), 1:1000.

3. Antibody validated in this study by APEX2 activation: Streptavidin Alexa-488 (S32354, Invitrogen)

4. Phospho-specific antibodies validated in this study as desribed in Figure 2 and 3, Extended Data figure 2 and 3: pTRF1-T358 and pTRF2-Ser65.

5. Antobodies validated by depletion as indicated on the publishers website: INCENP, 1:2000 (ab-12183, AbCam); Aurora B, 1:1000 (ab-2254, AbCam); Borealin (ab74473, Abcam), 1:1000; Survivin (NB500-201, Novus Biologicals).

6. Commonly used loading controls: beta-Actin (A5441, Sigma), 1:20,000; Actin (MAB1501R, Millipore), 1:10,000; Vinculin (V9131, Sigma), 1:5000; GAPDH (MAB374, Millipore).

7. Antibodies validated previously through controlled induction and suppression of a DNA damage response: γ-H2AX (Clone 2F3, Biolegend) 1:200 or (05-636, Merck Millipore) (Van Ly et al 2018, Molecular Cell; Masamsetti et al 2019 Nature Communications).

8. Commonly used commercial secondary antibodies: Goat anti-mouse HRP (P044701, DAKO Agilent), 1:5000; Goat anti-Rabbit HRP (P044801, DAKO Agilent); Alexa-568 goat anti-mouse (A11031, Invitrogen); 1:10,000

Eukaryotic cell lines

Policy information about cell lines and Sex and Gender in Research

Cell line source(s)	IMR90 fibroblasts (female) were purchased from Coriell Cell Repositories, and HCT116 (male) and HT1080 (male) cells from ATCC (American Type Culture Collection). HeLa (female) cells were provided by Megan Chircop (CMRI), HT1080 6TG (male) cells by Eric Stanbridge (University of California, Irvine), and TRF2Floxed/Floxed Rosa26-CreERT2 pBabeSV40LT MEFs (sex not determined) by Eros Lazzerini Denchi (National Cancer Institute). IMR90 E6E7 hTERT were created as described elsewhere (Romero-Zamora & Hayashi, 2023 Scientific Reports).
Authentication	Cell Bank Australia verified cell line identity using short-tandem-repeat profiling.
Mycoplasma contamination	All cells were routinely identified to be mycoplasma negative (MycoAlert, LT07-118, Lonza). IMR90 derivatives were cultured in Plasmocin (InvivoGen).
Commonly misidentified lines (See ICLAC register)	None of this cell lines in this study are included in the ICLAC register of misidentified lines.
